# Narrow-Band Imaging Colonoscopy to Assess Mucosal Angiogenesis in Ulcerative Colitis

**DOI:** 10.1155/2019/8312624

**Published:** 2019-02-24

**Authors:** Tao Guo, Jia-Ming Qian, Ai-Ming Yang, Yue Li, Wei-Xun Zhou

**Affiliations:** ^1^Department of Gastroenterology, Peking Union Medical College Hospital, Chinese Academy of Medical Sciences, Beijing 100730, China; ^2^Department of Pathology, Peking Union Medical College Hospital, Chinese Academy of Medical Sciences, Beijing 100730, China

## Abstract

**Background and Aim:**

It has been documented that angiogenesis is a largely unstudied component of the pathogenesis of ulcerative colitis (UC). Under narrow-band imaging (NBI) colonoscopy, the mucosal vascular pattern (MVP) can be visualized without the use of dyes. The aim of this study was to assess the grade of mucosal angiogenesis based on the MVP in UC.

**Methods:**

A total of 119 colorectal segments taken from 42 patients with UC were observed using NBI colonoscopy. The MVP was classified as follows: clear, obscure, or absent. Quantification of the degree of inflammation was performed using histological colitis scoring. Potent angiogenic activity was assessed by immunohistochemical staining for vascular endothelial growth factor (VEGF). Microvascular density was assessed using vessel counts as revealed by CD31 staining. The correlation between the MVP and histological grades of inflammation and angiogenesis was evaluated.

**Results:**

The MVP correlated well with the histological severity of inflammation. We also demonstrated an increasing level of microvascular density and VEGF staining along with the ordered types of MVPs. In addition, a statistically strong association existed between microvascular density and VEGF staining.

**Conclusions:**

NBI colonoscopy might be a useful tool for the *in vivo* assessment of the grade of mucosal angiogenesis in UC.

## 1. Introduction

Angiogenesis, the formation of new vasculature from an existing vascular network, may, in part, mediate the healing process and epithelial restitution. However, it has been suggested that angiogenesis plays a crucial role in various pathological processes, such as acute and chronic inflammation [1, 2]. Recent publications have demonstrated that altered angiogenesis may be a largely unstudied component of ulcerative colitis (UC) pathogenesis [3–5]. Vascular endothelial growth factor (VEGF) is the most potent angiogenic growth factor and has been shown to play a master role in promoting inflammation and regulating mucosal immune-driven angiogenesis in inflammatory bowel disease [6–8].

Narrow-band imaging (NBI) is an optical technique in which the applied light wavelengths, through the use of narrow-band filters, are restricted to those specific to hemoglobin absorption, thereby highlighting the intramucosal vascular network and mucosal surface structures without chromoscopy [9]. It has been proposed that NBI is useful for screening neoplastic lesions in long-standing UC [10]. To date, however, correlations between the NBI colonoscopy findings and the histological results associated with angiogenesis in UC have rarely been investigated.

In this study, we investigated whether NBI colonoscopy could be a useful tool to evaluate the grade of angiogenesis based on the mucosal vascular pattern (MVP) in UC patients.

## 2. Materials and Methods

### 2.1. Patients

This study was conducted at Peking Union Medical College Hospital between December 2012 and January 2015 using patients with an established diagnosis of UC. Patients who had proctitis, left-sided colitis, or pancolitis, and who had a wide range of activity across the Mayo score, were recruited for this study. The extent of UC was determined by colonoscopy, and the UC disease activity was assessed according to the Mayo score. Under a written informed consent for colonoscopy, patients were examined by NBI colonoscopy with biopsy. This study protocol conformed to the ethical guidelines of the 1964 Helsinki declaration and its later amendments and was reviewed and approved by the Ethics Committee of Peking Union Medical College Hospital (protocol number: S-K508).

### 2.2. Colonoscopy Procedure

Each patient underwent a colonoscopy with an endoscope (CF-H260AI; Olympus, Tokyo, Japan), using a prototype of the NBI system (Evis CV-260; Olympus, Tokyo, Japan). When performing an NBI colonoscopy, the endoscope was advanced into the cecum using the white-light endoscopy (WLE) mode. During withdrawal, a routine observation was performed, and the inflamed area was identified using the WLE mode. The colorectum was divided into six segments (cecum, ascending colon, transverse colon, descending colon, sigmoid colon, and rectum). For each segment possessing a lesion and immediately after identifying the inflamed area using the WLE mode, the imaging mode was switched to NBI with the resulting image recorded using an integrated image capture system and saved on a server for later assessment. The endoscope was required to be very close to the mucosa to acquire a satisfactory image under NBI mode. Subsequently, at least one mucosal biopsy specimen was obtained from the same area for histological assessment.

### 2.3. Image Assessment

To avoid possible selection bias and to maintain study quality, all NBI images from the target area were randomly allocated and evaluated by two trained endoscopists, who did not have access to the clinical data. The general consensus for each image was established by an assessment of the MVP and surface patterns, as described below.

**Figure 1:**
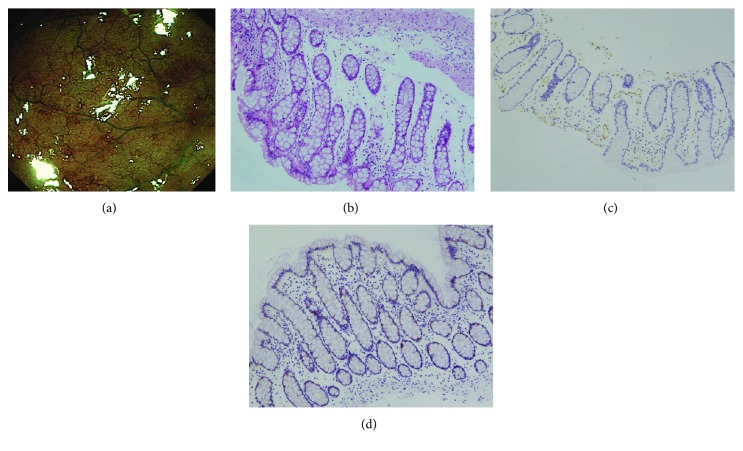
Endoscopic and histological findings of the mucosa classified as “clear pattern” under NBI. (a) Under NBI colonoscopy, the brownish intramucosal capillaries were clearly shown. (b) The histological degree of inflammation was judged to be mild. (c) Sporadic microvessels in the lamina propria and submucosa were revealed by immunohistological staining with endothelium marker CD31. (d) VEGF staining was predominantly localized in endothelial cells of vessel and in epithelial cells.

**Figure 2:**
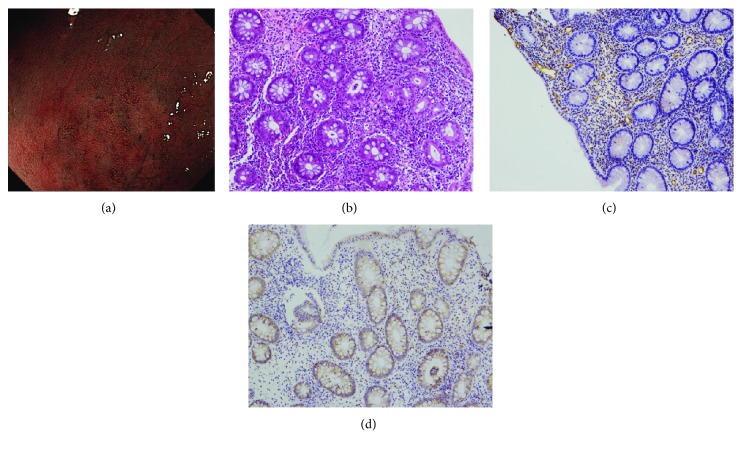
Endoscopic and histological findings of the mucosa classified as “obscure pattern” under NBI. (a) Under NBI colonoscopy, the brownish intramucosal capillaries were unclearly observed. (b) The histological degree of inflammation was judged to be moderate. (c) Increased microvessels in the lamina propria and submucosa were revealed by CD31 staining in comparison to “clear pattern.” (d) Increased VEGF staining in either endothelial cells or epithelial cells was observed in comparison to “clear pattern.”

**Figure 3:**
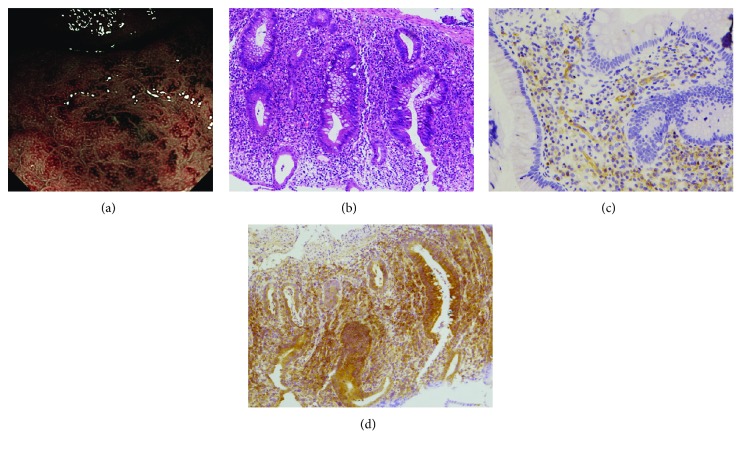
Endoscopic and histological findings of the mucosa classified as “crypt opening pattern” under NBI. (a) Under NBI colonoscopy, the intramucosal capillaries were invisible and round crypts were observed. (b) The histological degree of inflammation was judged to be severe. (c) Numerous microvessels in the lamina propria and submucosa were revealed by CD31 staining. (d) Numerous VEGF staining, in both endothelial cells and epithelial cells, was observed; in addition, infiltrating inflammatory cells were also stained for VEGF.

**Figure 4:**
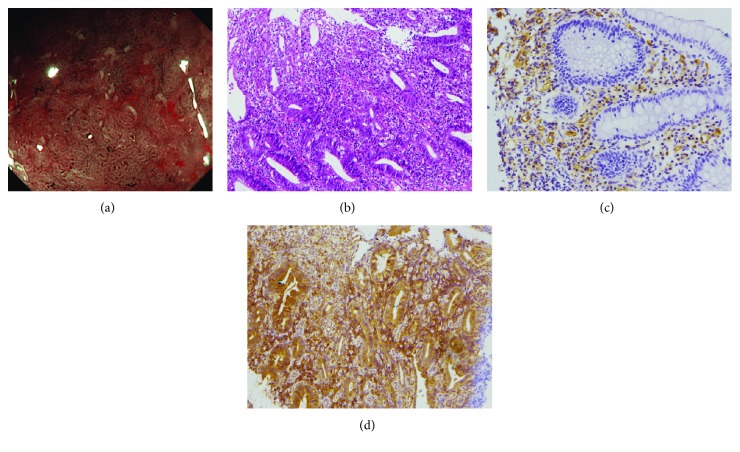
Endoscopic and histological findings of the mucosa classified as “villous pattern” under NBI. (a) Under NBI colonoscopy, the intramucosal capillaries were invisible and villous mucosal surface was observed. (b) The histological degree of inflammation was judged to be severe. (c) Numerous microvessels in the lamina propria and submucosa were revealed by CD31 staining. (d) Numerous VEGF staining in both endothelial cells and epithelial cells was observed; in addition, infiltrating inflammatory cells were also stained for VEGF.

When NBI colonoscopy demonstrated clear intramucosal capillaries, the MVP was defined as clear (Figure 1(a)). When NBI failed to reveal a clear vascular network or when the image was blurry, it was regarded as obscure (Figure 2(a)). In areas where the intramucosal vessels were invisible under NBI, the MVP was regarded as absent. Based on this protocol, the MVP patterns were classified into three types: clear, obscure, or absent. Since NBI enables observation of the surface patterns and the MVP, the surface pattern could be a viable assessment alternative during times when the MVP cannot be visualized under NBI. In this study, the absent pattern was subclassified into two types based on the observation of the surface pattern: a crypt opening pattern where NBI depicts whitish round crypts (Figure 3(a)) and a villous pattern where NBI shows villous structures (Figure 4(a)) [11].

### 2.4. Histological Assessment

All biopsy specimens were fixed in 10% formalin, embedded in paraffin, and stained with hematoxylin and eosin. Quantification of the degree of inflammation was determined using a histological colitis score from zero to four (0: no inflammation, 1: mild edema and inflammation in the lamina propria, 2: crypt abscess formation and inflammation in the lamina propria, 3: more severe inflammation with destructive crypt abscess, and 4: more severe inflammation with active ulceration) [12]. The histological grading score that we used had been validated and showed good correlations between microscopic scores and colonoscopic findings in patients with UC [12].

### 2.5. Immunohistochemistry

Immunohistochemical analysis for antigens was performed on 4 *μ*m sections using an Autostainer System (DakoCytomation, Carpinteria, CA) and a Ventana Benchmark XT Autostainer (Ventana Medical Systems Inc., Tucson, AZ). Two antibodies were used: CD31 (Dako) and VEGF (Zsbio Commerce Store, Beijing, China). CD31 was performed to highlight endothelin, and microvascular density was determined using vessel counts [13]. The extent of VEGF staining was assessed as the percentage of positively stained cells among 500 cells and classified into 5 categories (1: 0-5%, 2: 6-25%, 3: 26-50%, 4: 51-75%, and 5: 76-100%). The intensity of VEGF staining was graded as weak (1: +), moderate (2: ++), or strong (3: +++), using an arbitrary scale. Finally, a semiquantitative “VEGF staining index” was calculated by adding the percentage expression score to the staining intensity [14]. The histological variables were assessed by a pathologist who was blinded to the clinical and endoscopic findings.

### 2.6. Statistical Analysis

All statistical analyses were performed using the IBM SPSS Statistics 22 software package (IBM, New York, NY, USA). Wilcoxon Mann-Whitney *U* tests and Kruskal-Wallis tests were used for nonparametric values, and a one-way analysis of variance (ANOVA) was used for parametric values. For correlations, Spearman's rank correlation coefficient was used. *P* < 0.001 was used to indicate significant differences. The statistical methods of this study were reviewed by a biomedical statistician.

## 3. Results

### 3.1. Characteristics of the Study Subjects

A total of 42 patients with UC were included in the current study.  Table 1 summarizes the clinical features of the enrolled patients. Based on the colonoscopy and Mayo score, the disease extent and activity were determined.

### 3.2. NBI Colonoscopy Findings in Ulcerative Colitis

The MVP of 119 colorectal segments taken from 42 patients was assessed. Under NBI colonoscopy, 34 segments were determined to have a clear MVP, 58 segments were judged as having an obscure MVP, and 27 segments had an absent MVP. For the segments with a clear or obscure MVP, the surface pattern was evaluated as a crypt opening type. For the 27 segments with an absent MVP, the surface pattern was easily identified and determined as a crypt opening in 11 segments and as a villous pattern in 16 segments.

### 3.3. Correlation between the Degree of Inflammation and the Mucosal Pattern

Table 2 shows the relationship between the degree of inflammation and the different types of mucosal patterns. We observed that in clear patterns, 76.5% (26/34) of specimens showed only minor inactive inflammation, with an inflammatory score of 1. In obscure patterns, 75.9% (44/58) of specimens showed mild-moderate inflammatory activity, with an inflammatory score of 2-3. In the absent pattern group, nearly one-hundred percent of specimens showed moderate-severe inflammatory activity, with an inflammatory score of 3-4. The specimens with an obscure pattern showed a higher degree of inflammation compared to those with a clear pattern (*P* < 0.001). The degree of inflammation increased significantly (*P* < 0.001) in the absent pattern group compared to the obscure pattern group. However, when comparing the crypt opening pattern with the villous pattern, there was no significant difference in inflammatory activity (*P* = 0.309). As a whole, the ordered types of MVP correlated with the degree of inflammation (*P* < 0.001).

### 3.4. Comparisons of the Microvascular Density/VEGF Staining

CD31 staining was performed to detect microvessels. Samples from clear patterns showed sporadic thin vessels in the lamina propria and submucosa (Figure 1(c)), whereas tissues from obscure and absent patterns contained increased (Figure 2(c)) and numerous (Figures 3(c) and 4(c)) readily detectable dilated vessels in both the lamina propria and submucosa. After immunostaining, VEGF expression was found to occur in the cytoplasm and cell membrane. In samples with clear patterns, VEGF was predominantly localized in vessel endothelial cells as well as in epithelial cells (Figure 1(d)). VEGF staining of either cell type was increased in samples from obscure (Figure 2(d)) and absent (Figures 3(d) and 4(d)) patterns vs. clear patterns. Infiltrating inflammatory cells were also stained for VEGF.

The microvascular density in different types of mucosal patterns, as revealed by CD31 staining and the VEGF staining index, is presented in Table 3. We observed that there was a trend towards a growing level of microvascular density and VEGF staining along with the ordered types of mucosal vascular patterns. Compared to the clear pattern, the obscure pattern showed a significant (*P* < 0.001) increase in microvascular density (21.2 ± 7.4 vessels/field vs. 12.9 ± 4.4 vessels/field) and VEGF staining (5.1 ± 1.5 vs. 3.4 ± 1.2). Microvascular density (28.8 ± 9.4 vessels/field vs. 21.2 ± 7.4 vessels/field) and VEGF staining (6.4 ± 1.2 vs. 5.1 ± 1.5) were significantly (*P* < 0.001) higher in the absent pattern group than in the obscure pattern group. However, no statistically significant differences were found in microvascular density (27.8 ± 9.8 vessels/field vs. 29.4 ± 9.4 vessels/field, *P* = 0.711) or VEGF staining (6.3 ± 1.3 vs. 6.4 ± 1.2, *P* = 0.741) between the crypt opening pattern and the villous pattern groups. In addition, for all types of mucosal patterns, there was a significant (*P* < 0.001) positive relationship between microvascular density and VEGF staining.

## 4. Discussion

It has been shown that severe acute inflammation is closely associated with early recurrence of UC [15]. Thus, a practical histological procedure to identify active inflammation seems to be warranted in the management of the disease. A recent study has suggested that endocytoscopic NBI is a technique which can effectively evaluate inflammatory activity in UC [16]. However, the application of endocytoscopic NBI does have some limitations since only a small number of patients with UC can be evaluated using that procedure. The improved mucosal contrast provided by NBI colonoscopies have been successfully used to visualize angiogenesis and thereby detect areas of inflammation in UC more effectively when compared with standard WLE [17].

For the first time in this study, we classified the MVPs of colorectal segments from patients with UC into three pattern types (clear, obscure, or absent) using NBI colonoscopy. We found that the MVP correlated well with the histological severity of inflammation in UC. In the segment with histologically inactive inflammation (Figure 1(b)), a clear MVP was observed under NBI (Figure 1(a)). However, in the histological mildly-moderately inflamed mucosa (Figure 2(b)), the MVP, as seen with NBI, was obscure due to apparent edema and granularity (Figure 2(a)). In the segment with histological moderately-severely active inflammation, the inflamed mucosa was identified as a dark-brownish or black mucosa, with an invisible or absent MVP by NBI. Two types of surface patterns, a crypt opening pattern (Figure 3(a)) and a villous pattern (Figure 4(a)), were depicted in the mucosa with absent MVPs. However, we did not detect a difference in inflammatory severity when the two types were compared (Figures 3(b) and 4(b)).

It has been suggested that angiogenesis is an integral part of UC pathology [3–5]. The VEGF-mediated effects on endothelial permeability might play a predominant role in the pathogenesis of UC [6–8]. Here, we demonstrated that the level of microvascular density (Figures 1(c)–4(c)) and VEGF staining (Figures 1(d)–4(d)) significantly increased along with the ordered types of MVPs in UC patients. In addition, a statistically strong association exists between increased microvascular density and increased VEGF expression. However, between the two subclassifications of absent patterns (crypt opening and villous), we found no significant difference in microvascular density or in VEGF staining. A recent study has indicated that vascularization in UC is particularly prominent in areas of active inflammation, and angiogenesis and inflammation may become chronically codependent processes [18, 19]. Based on our findings, we postulate that increased angiogenic activity is associated with the severity of inflammation. VEGF-mediated angiogenesis might have proinflammatory effects due to an increasing vascular permeability, which causes vascular leakage and inflammatory cell infiltration, which, in turn, promotes the persistent inflammation seen in UC [20, 21].

This study has some limitations. Firstly, our study can only be considered as preliminary research due to the small number of subjects. Secondly, we did not compare the efficacy of an NBI colonoscopy with WLE in its ability to evaluate the degree of inflammation in UC. This was because the MVP of each segment with lesions was assessed via NBI colonoscopy in each patient while the standard Mayo endoscopic score was determined using WLE on the most severely inflamed area in each patient. Thirdly, the presence of ulcers or prominent spontaneous hemorrhaging on the surface mucosa is a major disadvantage during NBI assessment of MVPs; therefore, only a limited number of patients with severely active UC were recruited for this study.

In conclusion, our data suggest that NBI colonoscopy may be an easy and useful tool for evaluating MVPs and determining the severity of mucosal inflammation and angiogenic activity. Furthermore, it can help predict the subsequent clinical course in UC. To assess the clinical efficacies of the NBI colonoscopy, further prospective studies involving larger samples and direct comparisons with WLE are required.

## Figures and Tables

**Table 1:** Clinical features of the enrolled patients with UC.

Characteristics of patients	
Total number of patients	42
Sex	
Male	25
Female	17
Age (yr)	
Range	26-72
Median	45
Disease duration (yr)	
Range	3-19
Median	4.1
Extent of UC	
Pancolitis	8
Left-sided colitis	27
Proctitis	7
Disease activity based on Mayo score	
Inactive or quiescent (Mayo score: 0)	9
Mildly active (Mayo score: 1)	18
Moderately active (Mayo score: 2)	10
Severely active (Mayo score: 3)	5

UC: ulcerative colitis.

**Table 2:** Correlation between the degree of inflammation identified by histological colitis score and the mucosal pattern.

Degree of inflammation (histological colitis score)	Classification of mucosal pattern using NBI				
	Clear (*n* = 34)	Obscure (*n* = 58)	Absent (*n* = 27)	Absent (*n* = 27)	
				Crypt opening (*n* = 11)	Villous (*n* = 16)
0	0	0	0	0	0
1	26	14	0	0	0
2	8	28	1	1	0
3	0	16	11	5	6
4	0	0	15	5	10

NBI: narrow-band imaging.

**Table 3:** Comparisons of the microvascular density/VEGF staining in the mucosal patterns (mean ± SD).

	Classification of mucosal pattern using NBI				
	Clear (*n* = 34)	Obscure (*n* = 58)	Absent (*n* = 27)	Absent (*n* = 27)	
				Crypt opening (*n* = 11)	Villous (*n* = 16)
Microvascular density (vessels/field)	12.9 ± 4.4	21.2 ± 7.4^∗^	28.8 ± 9.4^#^	27.8 ± 9.8	29.4 ± 9.4
VEGF (staining index)	3.4 ± 1.2	5.1 ± 1.5^∗^	6.4 ± 1.2^#^	6.3 ± 1.3	6.4 ± 1.2

NBI: narrow-band imaging; VEGF: vascular endothelial growth factor. ^∗^*P* < 0.001*vs*. clear pattern. ^#^*P* < 0.001*vs*. obscure pattern.

## Data Availability

The data used to support the findings of this study are available from the corresponding author upon request.
